# Monitoring of Chemical Risk Factors for Sudden Infant Death Syndrome (SIDS) by Hydroxyapatite-Graphene-MWCNT Composite-Based Sensors

**DOI:** 10.3390/s19153437

**Published:** 2019-08-05

**Authors:** Narayanan Sudhan, Nehru Lavanya, Salvatore Gianluca Leonardi, Giovanni Neri, Chinnathambi Sekar

**Affiliations:** 1 Department of Bioelectronics and Biosensors, Alagappa University, Karaikudi-630 004, Tamilnadu, India; 2 Department of Engineering, University of Messina, 98122 Messina, Italy

**Keywords:** hydroxyapatite-based sensor, nicotine, caffeine, CO_2_, sudden infant death syndrome

## Abstract

Sensing properties of chemical sensors based on ternary hydroxyapatite-graphene-multiwalled carbon nanotube (HA-GN-MWCNT) nanocomposite in the detection of chemical substances representing risk factors for sudden infant death syndrome (SIDS), have been evaluated. Characterization data of the synthesized composite have shown that the graphene-MWCNT network serves as a matrix to uniformly disperse the hydroxyapatite nanoparticles and provide suitable electrical properties required for developing novel electrochemical and conductometric sensors. A HA-GN-MWCNT composite-modified glassy carbon electrode (HA-GN-MWCNT/GCE) has been fabricated and tested for the simultaneous monitoring of nicotine and caffeine by cyclic voltammetry (CV) and square wave voltammetry (SWV), whereas a HA-GN-MWCNT conductive gas sensor has been tested for the detection of CO_2_ in ambient air. Reported results suggest that the synergic combination of the chemical properties of HA and electrical/electrochemical characteristics of the mixed graphene-MWCNT network play a prominent role in enhancing the electrochemical and gas sensing behavior of the ternary HA-GN-MWCNT hybrid nanostructure. The high performances of the developed sensors make them suitable for monitoring unhealthy actions (e.g., smoking, drinking coffee) in breastfeeding women and environmental factors (bad air quality), which are associated with an enhanced risk for SIDS.

## 1. Introduction

Air pollution is well known as a potential risk factor for many diseases. A report on recent studies on air pollution exposure concluded that there exists evidence that air pollution also affects sudden infant death syndrome (SIDS), one of the leading causes of infant death during the first year of life which strikes approximately one in every 1000 babies worldwide [1]. Although reasons are still unknown, the rebreathing of exhaled carbon dioxide (CO_2_) trapped near an infant’s airway has been suggested as a possible reason for the increased SIDS risk [2,3]. It is well known that CO_2_ is an exogenous stressor, and so therefore an appropriate air quality standard for daycare facilities has been recommended. For this reason, children should not stay in a closed room during naptime in order to avoid high concentrations of this gas. The National Institute of Occupational Safety and Health (NIOSH) guidelines suggests that carbon dioxide concentration in indoor air above 1000 ppm is a clear indication of bad air, due to inadequate ventilation [4].

Recently, the combination of nicotine and caffeine has also received much attention due to their role in SIDS. Exposure of neonates to caffeine and nicotine is well known to be capable of affecting response to hypoxic conditions, a process thought to be related to SIDS [5,6]. These substances are found in high quantity in breast milk of women smoking and/or drinking coffee. Even if no recommended limits for these two substances are listed and it has been stated that low doses (<200 mg/day) of caffeine can be consumed during breastfeeding, the Food and DrugAdministration has advised pregnant and lactating women to avoid caffeine-containing foods and drugs if possible. Regarding nicotine, the American Academy of Pediatrics no longer lists nicotine as a drug that is contraindicated during breastfeeding [7].

However, during breastfeeding, these toxic substances pass to neonate, contributing to the development of an underlying vulnerability for SIDS. Heavy caffeine intake was found to be significantly associated with an increased risk in a case-control study [8]. Interestingly, caffeine and nicotine alone may not be toxic enough to create an underlying vulnerability for SIDS, but the combination of these factors are enough to put neonates at a higher risk of dying from SIDS [5]. 

The above chemical substances have been largely investigated for their adverse effects on human health. Nicotine is inhaled through cigarettes, while caffeine is introduced to the body by drinking coffee and soft drinks. These drugs do have certain positive effects, such as an increase in the level of acetylcholine in the brain [9,10,11], as well as negative effects such as elevated blood pressure, rapid breathing, heart palpitations, and insomnia due to extended periods under their influence [12]. CO_2_ is a gas present in ambient fresh air at a concentration of about 380 ppm. However, due to human and industrial activities and gas emissions from motor vehicles, such concentration increases notably. There is therefore an interest in developing simple, low cost, small, and reliable CO_2_ sensors for monitoring this gas in ambient air [13]. 

To our knowledge, this is the first report attempting to develop chemical sensors based on conductometric and electrochemical sensor platforms for monitoring these harmful substances. Among the variety of sensors, those used here are popular, cheaper, and with the largest commercial appeal. Their functioning mechanism and application has been reported in many reviews and books [14,15]. In the present work, we have first synthesized hydroxyapatite (HA, Ca_10_(PO_4_)_6_OH_2_)-graphene-multiwalled carbon nanotube nanocomposite (HA-GN-MWCNT) and used it as sensing material for (*i*) modifying glassy carbon electrode (GCE) for the electrochemical determination of nicotine and caffeine, and (*ii*) developing a conductometric CO_2_ gas sensor for monitoring indoor air quality (AQ). 

Hydroxyapatite is one of the major inorganic nanoporous biomaterials, attracting many interesting applications in the biomedical field due to its excellent mechanical properties and good biocompatibility, so it has several potential applications in bone and tooth replacement. HA has been also investigated as sensing material for chemical- and bio-sensors for both detection in liquid and gas environments [16,17,18]. The presence of H^+^ and OH^−^ ions is considered to be responsible for its ionic conductivity at room temperature, whereas oxygen ions (O_2_^−^, O^−^, etc.) play a key role at higher temperatures. However, for practical applications, conductivity of pure HA is not sufficiently high. A way to solve this limitation is to use HA composites, e.g., hydroxyapatite in conjunction with organic conductive fillers. Multi-walled carbon nanotubes (MWCNTs) and graphene (GN) have been largely used for this scope due to their positive mechanical properties and elevated electrical conductivity [19,20,21,22]. MWCNTs are promising candidate materials for chemical and biosensors due to their high surface areas for gas adsorption, provided by the central hollow cores and outside walls. The interest in graphene is attributed to the bearing of oxygen functional groups in edges and the basal plane of its honeycomb structure [23,24]. The combination of these two carbon materials offers a great flexibility and dispersion of MWCNTs on GO, due to the presence of oxygen-containing functional groups in GO nanosheets. In addition, this combination leads to an improvement in the electrical and electrochemical properties [25,26]. 

Interestingly, combining these two carbon nanostructures with HA resulted in a strong synergic effect and consequently led to a robust and superior composite material with improved electrical and electrochemical properties and reduced agglomeration which sustains the stability of the nanometric HA phase.

Herein, we make use of the advantages of HA-GN-MWCNT hybrid composite to detect the title toxic substances through a combination of electrochemical sensors for nicotine and caffeine and conductometric CO_2_ sensors. The developed sensors could be then used to monitor environmental risk factors for SIDS (e.g., smoking, drinking coffee) during breastfeeding and environmental risk factors (bad AQ) which are considered potential risk factors for the sudden infant death syndrome.

## 2. Materials and Methods

### 2.1. Reagents

Graphite powders were purchased from Sigma-Aldrich, while CTAB and MWCNT were purchased from Sisco Research Laboratory, Mumbai. Calcium nitrate tetra hydrate and di-ammonium hydrogen phosphate were purchased from Merk-Specialties, Mumbai. All chemicals were of analytical reagent grade and used without any further purification. 

### 2.2. Synthesis of Graphene Oxide

Graphene oxide (GO) sheets were prepared from graphite by a modified Hummer’s procedure [27]. In a typical experiment, 1 g of graphite powder was mixed with 50 mL of concentrated H_2_SO_4_ under stirring for 2 h. Then, 4 g of potassium permanganate was added at 30 °C for 1 h. Subsequently, deionized water (150 mL) was added, the temperature was raised to 90 °C, and the resultant mixture stirred for 30 min. Finally, 200 mL deionized water was added, followed by drop-by-drop addition of 3 mL of H_2_O_2_ (30%), changing the color of the solution from dark brown to greenish yellow. The precipitate obtained was separated from the solution, washed with deionized water, and finally dried under vacuum in order to obtain GO sheets.

### 2.3. Synthesis of HA-GN-MWCNT Composite

**Figure 1:**
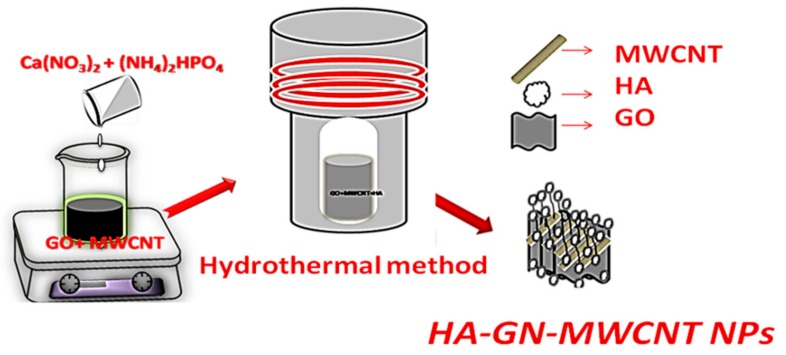
Representation of the procedure adopted for the synthesis of HA-GN-MWCNT composite.

HA-GN-MWCNT composites were synthesized through chemical precipitation method by the procedure reported in Figure 1.

Initially, 10 mL of the above synthesized GO colloidal solution (2 mg/mL) was subjected to ultrasonication treatment for 2 h. Subsequently, 20 mg of MWCNT was added into the GO solution and subjected to sonication for another 2 h. After the mixed solutions were centrifuged at 14,000 rpm for 30 min, the GO-MWCNT hybrid composite was obtained. 80 mg of Ca(NO_3_).4H_2_O was then added to the GO-MWNCT hybrid (20 mL) mixture, sonicated for 60 min, and then 40 mg of CTAB was added and sonicated for 60 min to form solution A. In another series, trisodium citrate (105 mg) and (NH_4_)_2_HPO_4_ (32 mg) were added to ultrapure water (15 mL) to form solution B which was poured into solution A. After that, the mixture was then kept in oven at 180 °C for 12 h and sonicated for 30 min. The composite was then washed with ethanol/water in order to remove unreacted reagents and dried for 24 h at 60 °C. The resultant product was named HA-GN-MWCNT.

### 2.4. Characterization

The samples morphology was investigated by SEM (ZEISS 1540XB FE SEM instruments, Zeiss, Germany) equipped with an EDX detector. The crystalline phases of the pristine HA and HA-GN-MWCNT composite were verified and compared by X-ray powder diffraction (XRD) using a XPERT-PRO diffractometer with Cu Kα radiation (1.5406 Å) at 40 kV. Fourier transform infrared (FTIR) was recorded from 4000 cm^−1^ to 400 cm^−1^ at room temperature with Thermo nichlolate instruments using pellet technique with KBr.

### 2.5. Electrochemical and Gas Sensing Tests

**Figure 2:**
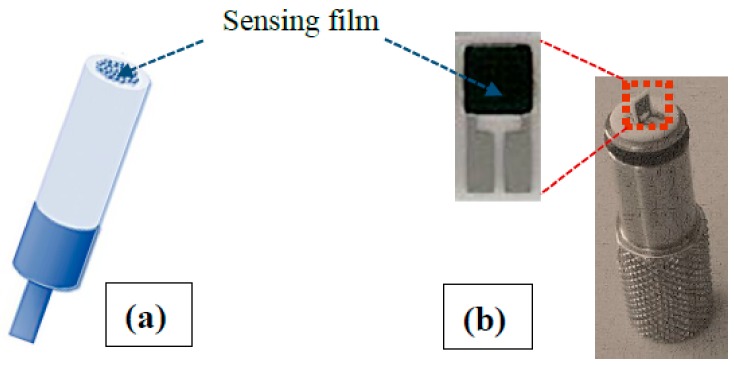
Picture of: (**a**) electrochemical and (**b**) conductometric sensor platforms. HA-GN-MWCNT composite sensing film area are indicated by arrows.

Electrochemical measurements were carried out on a CHI 609D workstation using a three-electrode system consisting of a platinum wire as a counter electrode, an Ag/AgCl as a reference, and the modified glassy carbon as working electrode (Figure 2a). Modified electrode was fabricated as follows: 5 mg HA-GN-MWCNT composite was dissolved in deionized water (1 mL) and sonicated for 30 min at room temperature to obtain homogenous black colored dispersion. 10 µL of the dispersion was dropped onto the surface of the carefully polished glassy carbon electrode by using 0.1, 0.3, 0.5 µm alumina powder. For comparison, we prepared pristine HA, GO, and MWCNT modified electrodes by following the same procedure.

Resistive sensors were fabricated mixing the samples with water to obtain a paste and printing a thick film (~10 µm) on alumina planar substrates (3 mm × 6 mm) supplied with interdigitated Pt electrodes (Figure 2b). A heating element, present on the back side, was used to set the operating temperature of the sensor. Sensing tests were performed at a controlled temperature, varying the CO_2_ concentration in humid synthetic air (total stream 100 sccm). Measurements were carried out, recording the sensors’ resistance by means of a multimeter (Agilent 34970A, Santa Clara, CA, USA). An air bubbler was used to generate a controlled relative humidity (RH) atmosphere. The sensor response, S, is defined as R-R_0_/R_0_, where R and R_0_ are the sensor resistance in air and in the presence of CO_2_, respectively.

## 3. Results and Discussion

### 3.1. Synthesis and Characterization of HA-GN-MWCNT Composite

HA-GN-MWCNT ternary composite was synthesized by the chemical precipitation method (see Figure 1) reported in detail in the experimental section. This procedure had been adopted previously for synthesizing HA-graphene composite for tissue engineering applications [28]. As already reported by other authors, the oxygen-functional groups on GO and MWCNTs surfaces may behave as receptor sites for Ca^2+^ through electrostatic interactions. Thus, anchored Ca^2+^ ions can react in-situ with the phosphate ions giving composite nanoparticles with a high degree of interfacial bonding [29]. Further, a reduction of the GO to GN occurs due to the presence in the reaction mixture of sodium citrate, a well-known reducing agent [30]. These processes lead then to the easy formation of the HA-GN-MWCNT ternary composite.

**Figure 3:**
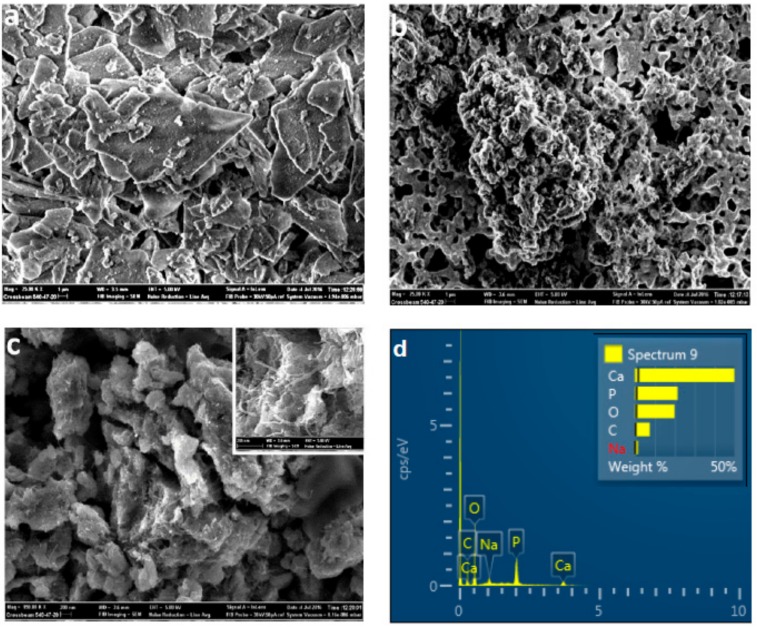
SEM images of (**a**) pure HA and (**b** and **c**) HA-GN-MWCNT. (**d**) EDX analysis of the HA-GN-MWCNT composite sample.

The surface morphology of the HA-GN-MWCNT composite was investigated by SEM (Figure 3a–c). The typical sheet morphology of the pristine HA is highlighted first at low magnification (scale bar is 1 μm) in Figure 3a. Image at this magnification of HA-GN-MWCNT composite (Figure 3b) shows that the hybrid sample is highly porous and is composed of HA nanoparticles dispersed uniformly onto the graphene sheets.

At higher magnification (scale bar 200 nm) MWCNTs can be easily imaged (inset in Figure 3c provide better details for this). EDX elemental analysis (Figure 3d) shows the presence of Ca, P, and O elements, typical of HA and C coming from graphene and MWCNTs. Based on SEM observations, it appears that the graphene oxide sheets act as a substrate to support and disperse HA, whereas CNTs act as bridges between them.

**Figure 4:**
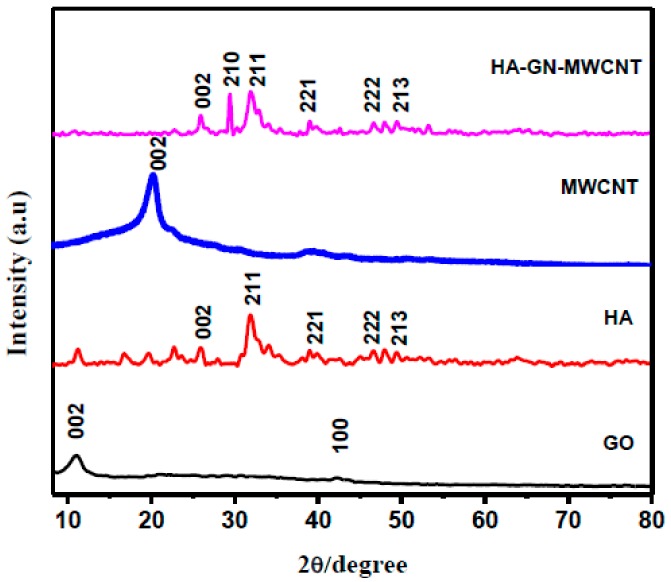
XRD patterns of HA-GN-MWCNT composite sample and single components.

Powder X-ray diffraction patterns of GO, HA, MWCNT, and HA-GN-MWCNT composite are shown in Figure 4. The reported XRD patterns of single components are in agreement with reference standard compounds. Diffraction peaks of HA-GN-MWCNT composite are also in agreement with the ICDD (JCPDS#09-432) values of standard HA crystallites. Of note, reflection related to 210 plane is strongly enhanced in intensity, indicating a possible orientation of the HA formed on the graphene sheets during the synthesis procedure. The absence of 2θ peak characteristics of GO at 10.92-theta degree confirms the reduction of raw GO to graphene [27]. However, no trace of reflections related to graphene and MWCNTs was detected in the composite sample. These findings can be explained on the basis of a strong interaction occurring between the in-situ formed HA and graphene sheets [31]. 

**Figure 5:**
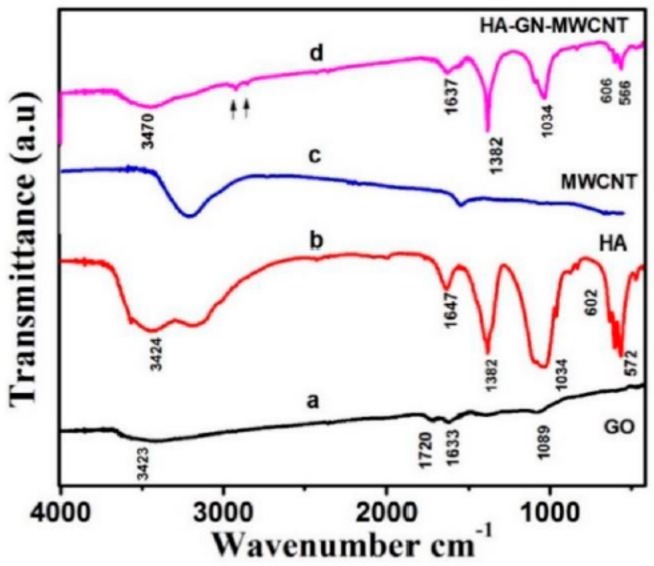
FT-IR of HA-GN-MWCNT composite sample and single components.

FT-IR analysis has been carried out to acquire additional information on the microstructure and functional groups present in the individual components and the ternary composite (Figure 5). The oxygen-containing functional group characteristics of GO were noticed on the raw graphene oxide by bands at 1089, 1633, and 1720 cm^−1^, assigned to the stretching vibrations of C–O, C–OH, C=C, and C=O in the COOH group [29]. The broad band around 3423 cm^−1^ is attributed to the stretching mode of the O–H group. MWCNTs pattern exhibits a broad band of O–H group around 3443 cm^−1^ and a weak peak at 1780 cm^−1^, indicating an oxidation of the walls. HA pattern is dominated by absorption bands at 1034, 572, and 602 cm^−1^, attributed to PO_4_^3−^ group in HA.

FT-IR spectrum of the HA-GN-MWCNT composite clearly shows the characteristics absorption bands related to hydroxyapatite. The FT-IR spectra also confirms the presence of graphene sheets in the composite as demonstrated by the appearance of absorption bands attributed to the symmetric (sCH_2_) and asymmetric (aCH_2_) at around 2853 cm^−1^ and 2926 cm^−1^ (see arrows in the FT-IR spectrum of composite), respectively. The absence of band at 1720 cm^−1^ and the characteristics of raw GO further give confirmation to the reduction process occurring during synthesis of the composite sample. The small band at 1600 cm^−1^ is related to the presence of MWCNTs. These findings confirm that the ternary composite has the composition HA-GN-MWCNT.

### 3.2. Electrochemical Behavior of HA-GN-MWCNT/GCE

**Figure 6:**
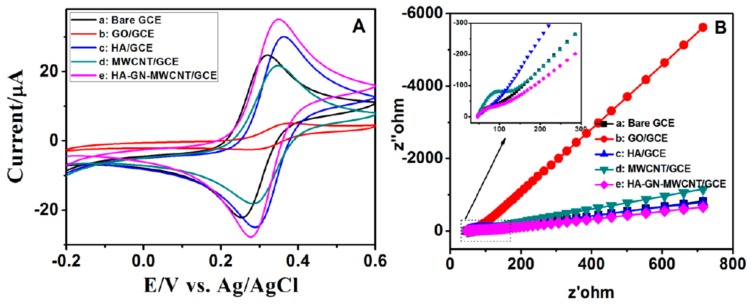
(**A**) CVs of (**A**) bare GCE, (b) GO, (c) HA, (d) MWCNT, (e) HA-GN-MWCNT modified GCEs in 1 mM [Fe(CN)_6_]^3−/4−^ at a scan rate of 50 mV/s. (**B**) Electrochemical impedance spectra of the corresponding electrodes recorded at the DC potential of 200 mV, AC potential ±5 mV.

The HA-GN-MWCNT composite was used to modify glassy carbon electrodes. A detailed description of the fabrication procedure of modified GCEs can be found in a previous paper [32]. The experimental data and the related discussion for the detection of nicotine and caffeine are reported below. The electrochemical properties of HA-GN-MWCNT-modified GCE were evaluated by cyclic voltammetry (CV). Figure 6A shows CV cycles of different modified electrodes recorded in the presence of 1 mM [Fe(CN)_6_]^3−/4−^ in −0.1 M KCl.

Well-resolved anodic and cathodic peaks are observed for all the investigated electrodes. The separation potential (ΔE) of the two redox peaks for HA-GN-MWCNT/GCE and HA/GCE are 71 and 92 mV, respectively. A fast electron transfer process is suggested by the lower value of ΔE. To study the interface properties of surface-modified electrodes, EIS was recorded with a frequency ranging from 100 kHz to 1 Hz at the DC potential 200 mV, AC potential ±5 mV in presence of 0.1 M KCl containing 1 mM [Fe(CN)_6_]^3−/4−^. The Nyquist plots of modified electrodes are shown in Figure 6B. The semicircle diameter of EIS equals the charge transfer resistance R_CT_. The R_CT_ values for (a) bare GCE, (b) GO, (c) HA, (d) MWCNT and (e) HA-GN-MWCNT modified GCEs were estimated as 40, 485, 376, 138, and 47 Ωcm^−2^ respectively. It was obvious that pure GO modified GCE had the highest R_CT_ value while, on the contrary, electron transfer rate of HA-GN-MWCNT/GCE was higher than all the other electrodes. These observations suggest that the ternary composite had a higher electrocatalytic activity than the pristine HA and GO modified GCEs.

#### 3.2.1. Electrochemical Determination of Nicotine and Caffeine 

**Figure 7:**
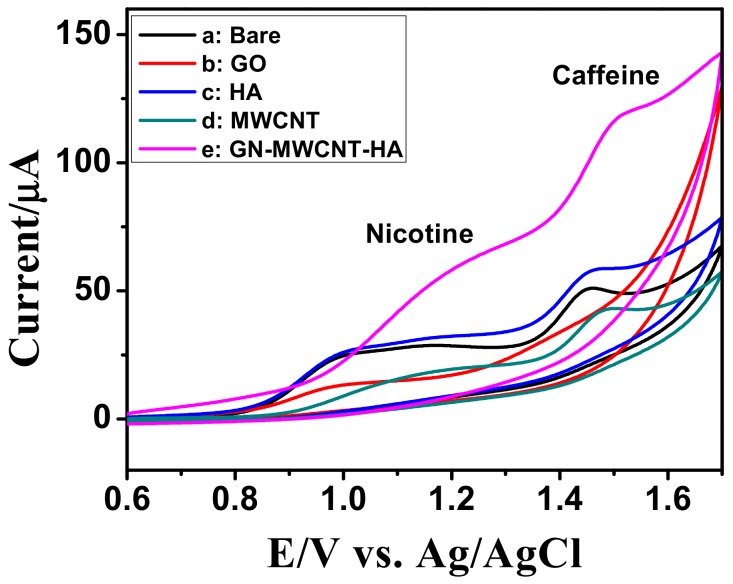
CVs obtained for the mixture of 1mM of nicotine and caffeine in 0.1 M PBS (pH 7.0) at modified GCEs at a scan rate of 50 mV s^−1^.

The electrocatalytic oxidation of nicotine and caffeine on HA-GN-MWCNT/GCE was investigated by cyclic voltammetry. Figure 7 shows the CVs recorded at different modified electrodes for 1 mM mixture of nicotine and caffeine in 0.1 M PBS (pH 7.0) at 50 mVs^−1^.

Among all the investigated electrodes, the I_pa_ value of HA-GN-MWCNT composite was found to be much higher with a slight positive shift in their peak potentials, indicating that the composite greatly enhance the electron transfer process of nicotine and caffeine simultaneously.

#### 3.2.2. Selective Determination of Nicotine and Caffeine 

**Figure 8:**
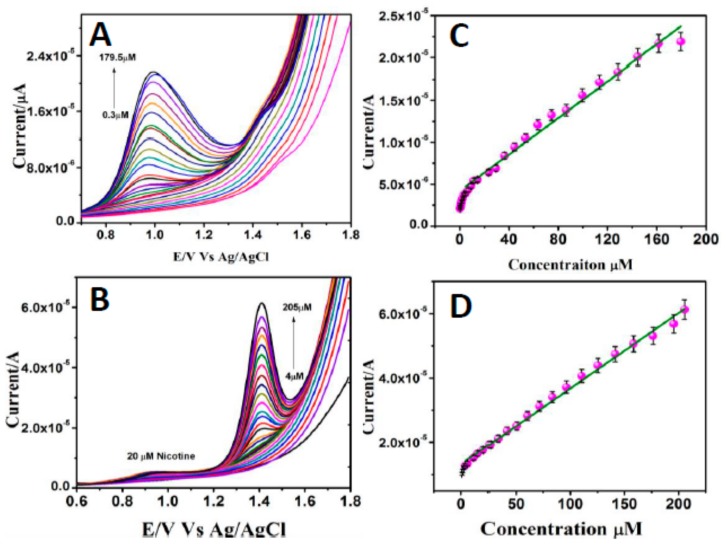
SWVs obtained for the various concentrations of (**A**) nicotine (0.3-179.5μM) in the presence of 75μM of caffeine and (**B**) caffeine (4-205μM) in the presence of 20μM of nicotine in 0.1 M PBS (pH 7.0) at HA-GN-MWCNT/GCE. In (**C**) and (**D**) are shown the corresponding calibration curves.

The sensing ability of the HA-GN-MWCNT/GCE for selective determination of nicotine and caffeine was investigated by SWV method in 0.1 M PBS (pH 7.0). Error bars are standard errors from triplicate measurements. The quantification of these analytes had been deduced from measurements carried out in triplicate. As shown in Figure 8A, the oxidation peak currents increased with the increasing concentrations of nicotine in the presence of 75 µM of caffeine, while oxidation peak potentials remained steady under constant stirring. The same behavior was observed for caffeine (Figure 8B). Linear calibration plots were obtained for nicotine (Figure 8C), in the range of 0.3 to 179.5 µM with a LOD of 0.21 µM, and caffeine (Figure 8D). The lowest limit of detection estimated for caffeine was 1.42 µM.

#### 3.2.3. Simultaneous Determination of Nicotine and Caffeine 

**Figure 9:**
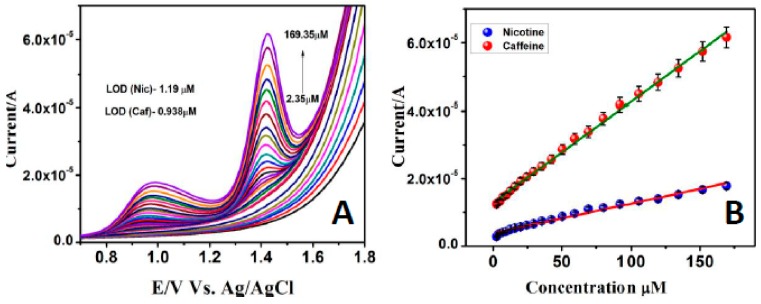
(**A**) SWVs obtained for various concentrations of nicotine and caffeine (2.35 to 169.35 μM) at HA-GN-MWCNT modified GCE in 0.1 M PBS (pH 7.0). (**B**) calibration curves.

To inspect the leeway of using the HA-GN-MWCNT/GCE for simultaneous determination of nicotine and caffeine, the anodic peak current responses at different concentrations in the mixture had been measured by the SWV method in 0.1 M PBS (pH 7.0). It can be seen from (Figure 9A) that the peak currents increased proportionally with concentrations over the wide linear range of 2.35–169.35 µM for both nicotine and caffeine and the lowest detection limits were deduced as 1.19 µM (nicotine) and 0.938 µM (caffeine), respectively. 

The linear regression equations for nicotine and caffeine were obtained through the calibration curves (Figure 9B) deduced from measurements carried out in triplicate, as I_pa_ = 1.9808C_Nicotine_ + 6.305 (R^2^ = 0.98405) and I_pa_ = 1.3305C_Caffeine_ + 2.345 (R^2^ = 0.99483). These results demonstrated that the high surface area and trapping ability of ternary composite offered a good electrocatalytic activity towards simultaneous detection of these two alkaloid compounds with clinical importance. Moreover, HA-GN-MWCNT composite preparation method of the present study is simple and reproducible.

#### 3.2.4. Interference and Reproducibility Studies

The anti-interference ability of the HA-GN-MWCNT/GCE was investigated by evaluating its response to the nicotine and caffeine mixture in the presence of 100-fold excess of several common co-existing substances such as xanthine, hypoxanthine, uric acid, L-tryptophan, ascorbic acid, and glucose. No obvious oxidation peaks corresponding to these interferents were observed. However, 100-fold excess of theophyline grants a small anodic peak close to the oxidation potential of nicotine. No remarkable change in the peak currents was noted in the presence of the interfering species, proving that the HA-GN-MWCNT/GCE sensor has good electrochemical recognition quality for nicotine and caffeine. For evaluating the reproducibility of the HA-GN-MWCNT/GCE, five repeated measurements were done for two alkaloids at the same electrode. The relative standard deviation (RSD) of 2.67% revealed that the fabricated sensor had good reproducibility.

#### 3.2.5. Real Sample Analysis

**Figure 10:**
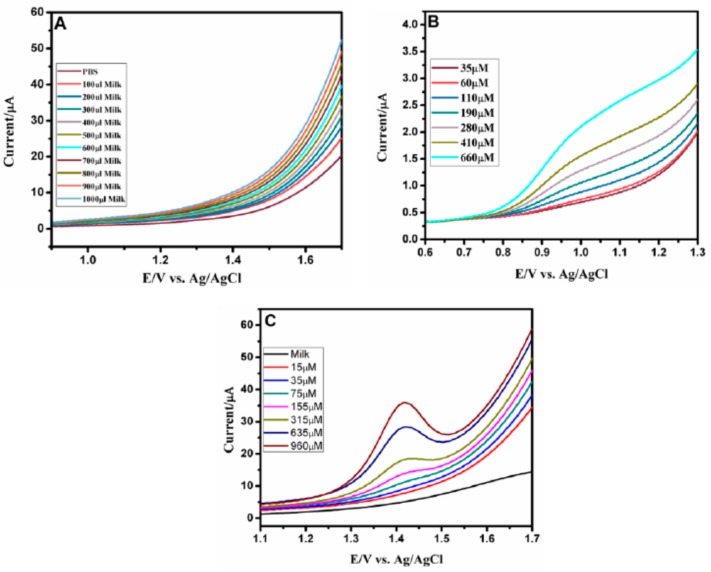
SWVs for determination of nicotine and caffeine in milk samples with: (**A**) without spiking of nicotine and caffeine at HA-MWCNT-GN/GCE in 0.1MPBS at pH 7.0. (**B**) Standard addition of known concentration of nicotine from 35 µM to 660 µM at HA-MWCNT-GN/GCE in 0.1MPBS at pH 7.0. (**C**) Standard addition of known concentration of caffeine from 15 µM to 950 µM at HA-MWCNT-GN/GCE in 0.1MPBS at pH 7.0.

Because smoking and/or consuming caffeine contribute to the development of an underlying vulnerability for SIDS, it is important to evaluate the presence of these substances in mother milk. However, not having the possibility to perform tests with mother milk, in order to evaluate the adaptability of this sensor to detect the desired analytes in a real complex sample, we tested the determination of caffeine in commercial cow milk (see Figure 10). 

Milk samples were utilized without any pretreatment using SWV method. Before, we tested the possible presence of nicotine and caffeine in these milk samples without the spiking of synthetic samples Figure 10A. There were no obvious peak currents observed for nicotine and caffeine in this analysis, which implies that there is no amount of nicotine and caffeine in milk samples. The developed sensor was then tested to detect nicotine and caffeine in milk samples by standard addition method. Real sample solutions were prepared by adding known concentrations of nicotine and caffeine in milk samples without any pre-treatment. SWV response was measured after the addition of different known concentrations of nicotine and caffeine into the milk sample in 0.1M PBS at pH 7.0 (Figure 10B,C).

### 3.3. HA-GN-MWCNT Conductometric Sensor for Environmental CO_2_

The as-prepared HA-GN-MWCNT ternary composite was used to fabricate conductometric CO_2_ sensors. A detailed description of the fabrication procedure and sensing platform of this typology of sensors can be found in previous papers [33]. Monitoring CO_2_ with simple conductometric sensors is an important issue in the field of environmental monitoring. A key aspect in this regard is the search for suitable sensing materials. Recently, we have shown that nanomaterials based on ZnO and containing calcium display better performance toward CO_2_ compared to pristine ZnO due to the enhanced absorption of CO_2_ provided by Ca basic sites [34,35]. Although ZnO-Ca sensors display high sensitivity, they operate at a high temperature (300–450 °C), while CO_2_ sensors for advanced applications are required to function with reduced power consumption, e.g. operating at low temperatures as possible, and which can be portable and powered by a battery [36]. 

As the hydroxyapatite is rich in Ca, it appears suitable as sensing material for developing CO_2_ conductometric sensor. This idea is confirmed in previous studies reported in literature by the research group of Mahabole and collaborators [37]. However, while we attempted to use pure HA for conductometric CO_2_ sensor operating at a low temperature (max 150 °C), the baseline resistance resulted out of the measurement range of our instrumentation (>200 MΩ), precluding any investigation. HA-GN-MWCNT composite instead possessed the required electrical characteristics to be investigated as a CO_2_ sensor at low temperature. Indeed, the presence of highly conductive GN and MWCNTs in the composite resulted in a strong decrease of the baseline resistance (around 1 KΩ in humid air in the temperature range between 125–150 °C). 

#### Conductometric Tests with HA-GN-MWCNT for CO_2_ Monitoring 

**Figure 11:**
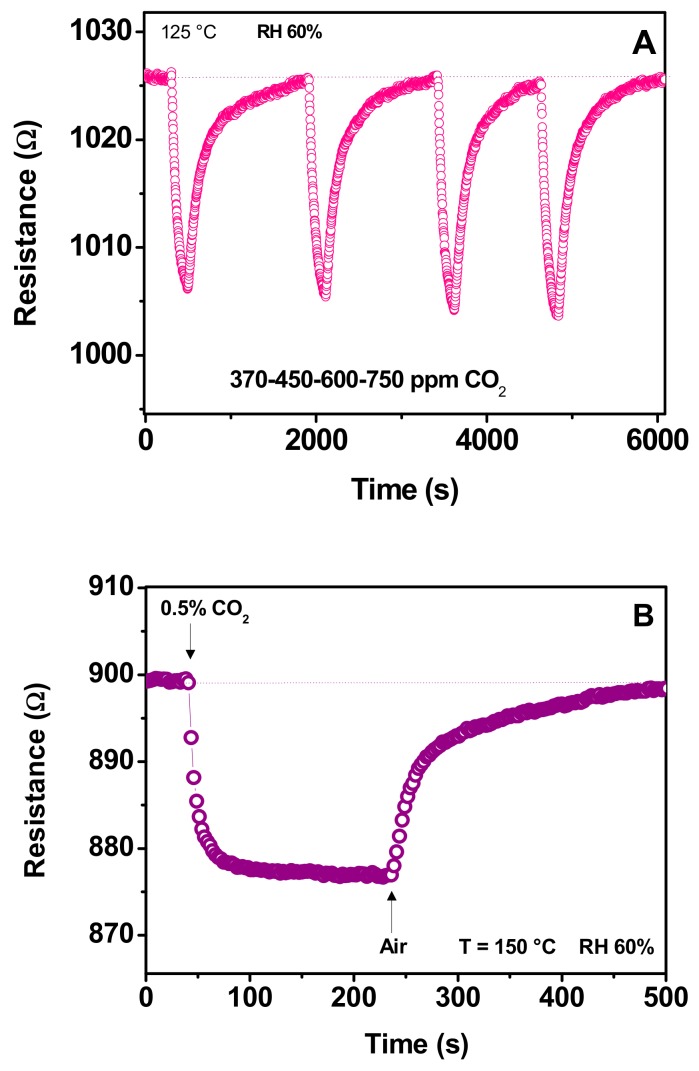
Transient response of the sensor to pulses of CO_2_ at the working temperature of (**A**) 125 °C and (**B**) 150 °C.

Figure 11 shows the transient response of the sensor at the working temperatures of 125 °C (Figure 11A) and 150 °C (Figure 11B) exposed to CO_2_ pulses of the duration of 200 s. At the lower temperature tested, the sensor responded to CO_2_ with a decrease of the resistance, but showed a slow recovery.

On the basis of previous data and the decrease of resistance observed, we assume that the above behavior arose from the reaction of CO_2_ with adsorbed OH^−^ present on the surface sensing material to form carbonates and hydroxyl carbonates. The key role of these adsorbed species in the sensing mechanism of the Ca-containing sensing layer had been reported by other authors. Morante and coworkers reported in-situ FT-IR measurements, demonstrating that Ca added to mesoporous In_2_O_3_ favors the interaction between surface CO_3_^2−^ and CO_2_ with the assistance of water, yielding bicarbonate products [38]. These adsorbed species then reacted with oxygen presence on the sensing material surface, releasing electrons in the bulk which, consequently, led to a decrease of the resistance. 

**Figure 12:**
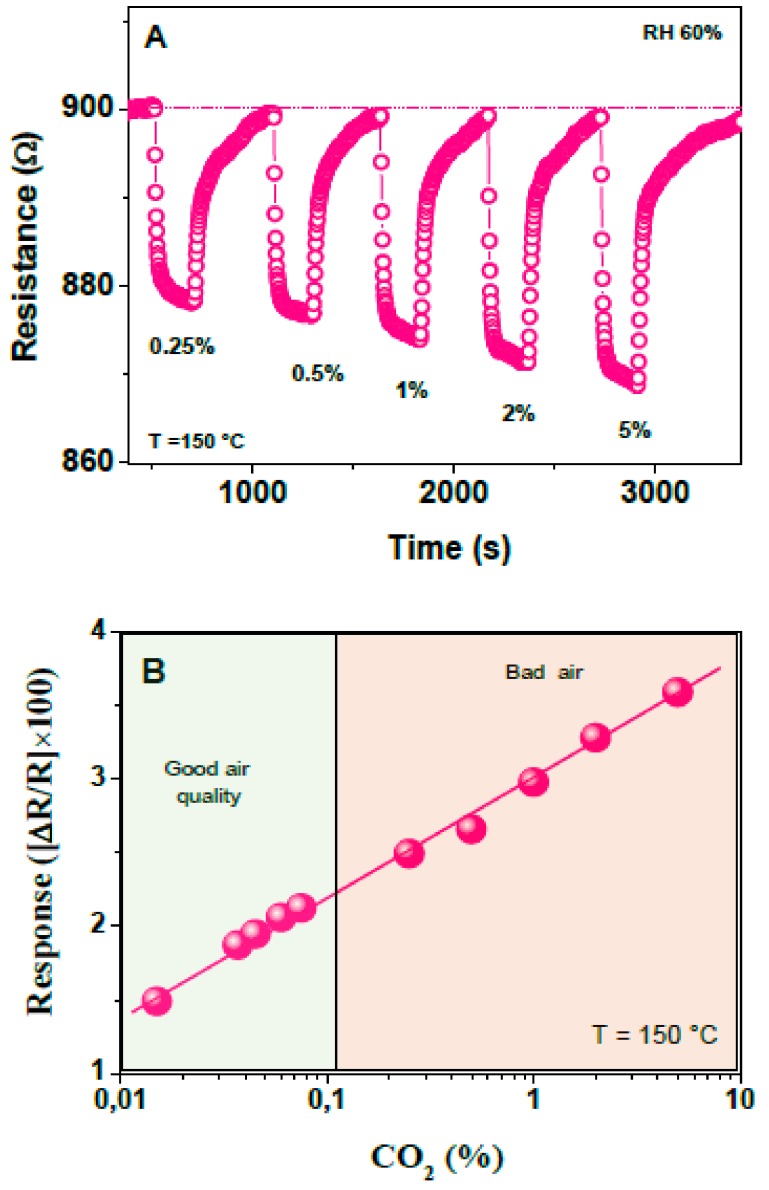
(**A**) Transient response of the sensor at the working temperature of 150 °C exposed to CO_2_ pulses of different concentration, ranging from 0.25 to 5%; (**B**) Calibration curve of the investigated sensor in a wide range of concentration (0.015 to 5%).

The ability of the sensor, operating at the temperature of 150 °C, to detect larger CO_2_ concentrations, ranging from 0.25% to 5%, is also reported in Figure 12A. The related calibration curve is shown in Figure 12B, where it can be noted the wide dynamic range covered, from less than the CO_2_ concentration present in fresh ambient air (around 380 ppm) to some percent. As a guide, the CO_2_ concentration that is permissible in the ambient maintaining good air quality (i.e. less than 0.1% or 1000 ppm), is highlighted in green. In brown is highlighted the region of higher CO_2_ concentration determining bad air quality.

Tests with different gases, i.e., CO, NH_3_, ethanol, and H_2_, which can be considered as interferent gaseous species in the monitoring of CO_2_ in ambient air have also been performed (not shown). Very little or no variation of the electrical resistance was observed during the functioning of the sensor in the experimental conditions above reported, indicating a high selectivity of the HA-GN-MWCNT conductometric sensor for CO_2_.

The above test demonstrated that the HA-GN-MWCNT sensor has the required performances for monitoring CO_2_ present in a closed ambient environment such as a bedroom, where base CO_2_ levels are expected to increase as a result of human occupancy and could exceed that recommended for children to safely sleep [4,39].

## 4. Conclusions

In the present work, a HA-GN-MWCNT composite was prepared by simple chemical precipitation method. The ternary composite was utilized for the simultaneous electrochemical determination of nicotine and caffeine by square wave voltammetry method. The proposed HA-GN-MWCNT/GCE showed high selectivity and sensitivity for individual and simultaneous determination over a wide linear range from 2.35–169.35 µM with the lowest detection limits of 1.19 and 0.938 µM for nicotine and caffeine, respectively. Moreover, the fabricated sensor has been used for determination of caffeine in a real sample with good accuracy. The ternary composite HA-GN-MWCNT was also used for developing a solid-state gas sensor which showed high performance for detecting low concentration of CO_2_ in ambient air.

The results suggest that the synergic action between chemical properties of HA and the mixed graphene-MWCNT network is fundamental in enhancing the electrochemical and gas sensing characteristics of the HA-GN-MWCNT hybrid nanostructure. These very simple sensors could be useful in assessing the risk factors of sudden infant death syndrome (SIDS) by detecting key biomarkers and using them to control both the quantity of nicotine and caffeine in mother milk and CO_2_ in the bedroom.
